# Delayed discovery, dissemination, and decisions on intervention in environmental health: a case study on immunotoxicity of perfluorinated alkylate substances

**DOI:** 10.1186/s12940-018-0405-y

**Published:** 2018-07-31

**Authors:** Philippe Grandjean

**Affiliations:** 1 Department of Environmental Health, Harvard T.H. Chan School of Public Health, Boston, MA USA; 2 Department of Environmental Medicine, University of Southern Denmark, Odense, Denmark

## Abstract

Identification and characterization of environmental hazards that impact human health must rely on the best possible science to inform and inspire appropriate public health intervention. The perfluorinated alkylate substances (PFASs) are persistent emerging pollutants that are now being recognized as important human health hazards. Although the PFASs have been produced for over 60 years, academic research on environmental health aspects has appeared only in the most recent 10 years or so. In the meantime, these persistent chemicals accumulated in the global environment. Some early studies e.g., on population exposures and toxicity, were not released to the public until after year 2000. Still, the first PFAS risk assessments ignored these reports and relied on scant journal publications. The first guidelines and legal limits for PFAS exposure, e.g., from drinking water, were proposed 10 years ago. They have decreased substantially since then, but remain higher than suggested by data on human adverse effects, especially on the immune system, that occur at background exposure levels. By now, the best-known PFASs are being phased out, and related PFASs are being introduced as substitutes. Given the substantial delays in discovery of PFAS toxicity, in dissemination of findings, and in regulatory decisions, PFAS substitutes and other persistent industrial chemicals should be subjected to prior scrutiny before widespread usage.

## Late emergence of early evidence

Industrial chemicals are often regarded inert or safe, unless proven otherwise, i.e., the so-called “untested chemicals assumption,” although this belief is of course not logical [1, 2]. A high-priority group of environmental chemicals, the perfluorinated alkylate substances (PFASs), constitute a clear example how narrow reliance on published toxicity studies can be misleading and result in insufficient and delayed protection of public health [3]. New insight on PFAS immunotoxicity shows that the path from discovery of toxicity to decisions on intervention can be stalled for decades (Table 1).

**Table 1:**
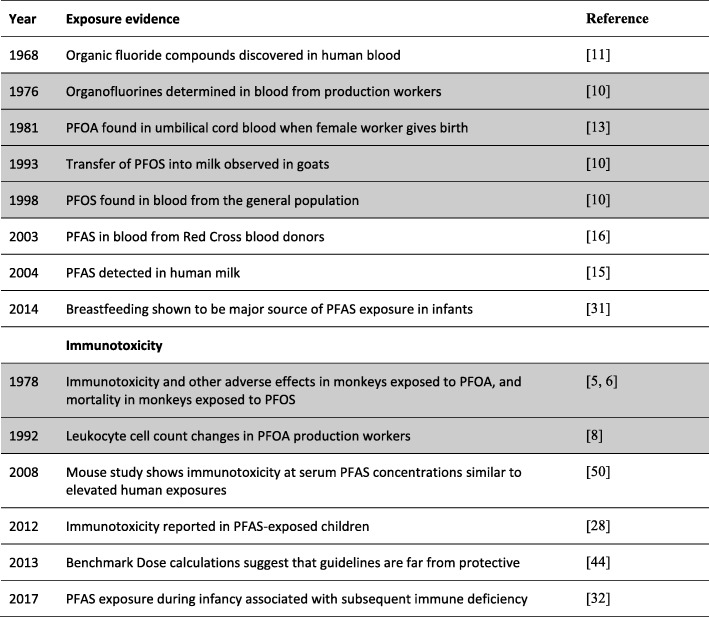
Time course of important developments regarding PFAS exposure and health risks [5, 6, 8, 10, 11, 13, 15, 16, 28, 31, 32, 44, 50]

Unpublished information is shaded

After the beginning of commercial PFAS production in the 1950s, a brief review article from 1980 [4] for the first time mentioned industry-sponsored studies, some of which were carried out in monkeys. Perfluorooctanoic acid (PFOA) showed specific toxicity to the reticuloendothelial system (i.e. immune system) [5]. In this 90-day study, compound-related microscopic lesions were seen in bone marrow, spleen and lymph nodes, thus clearly suggesting immunotoxicity, although functional tests were not carried out. A parallel study on perfluorooctanoic sulfonic acid (PFOS), also from 1978, was aborted due to mortality of the monkeys at all doses (the lowest being 10 mg/kg/day) [6]. These two internal reports were eventually shared with the U.S. Environmental Protection Agency (EPA) in 2000 [7] and then became accessible to the public.

A medical thesis from 1992 mentioned the evidence from the monkey study and noted: “No follow-up studies of these observations have been reported” [8]. The thesis analyzed clinical examination data from PFOA production workers and found clear associations between increased PFAS concentrations in the blood and decreased leukocyte counts. The results were not reported in a scientific journal. However, in connection with a recent law suit, a draft manuscript on this study has been released (“Peripheral blood lymphocyte count in men occupationally exposed to perfluorooctanoic acid” [9]). The draft concluded: “PFOA is associated with alterations in peripheral blood lymphocyte numbers in PFOA production workers, suggesting that cell-mediated immunity may be affected by PFOA”. Other company materials outlined in an expert report include the comment “We’re working with [the author] regarding some of the wording” [10]. Evidently, an agreement was not reached, and the findings were not published.

Human exposure to organofluorine compounds was discovered as early as 1968 [11] and was later confirmed in a more extensive study [12]. However, the exact identity and the sources were unknown at the time. Soon thereafter, PFASs were identified in blood from production workers, and in 1981 also in umbilical cord blood at a female worker’s childbirth [13]. Although the latter finding signified placental passage and prenatal PFAS exposure, this observation was not revealed until 20 years later, after which it was soon confirmed in a larger study [14]. Of additional public health significance, an unpublished study on goats from 1993 showed that PFOS was transferred into milk [10], and this pathway was verified in humans, again many years later [15].

## New insight into a hidden hazard

**Fig. 1:**
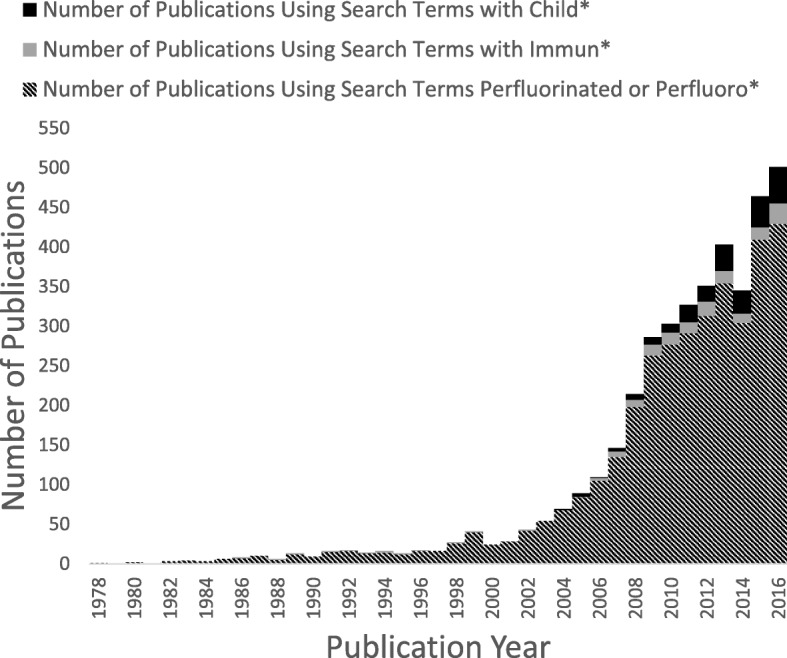
Number of publications on PFASs over time, according to the Web of Science database (between 1978 and 2017), using the search terms “perfluorinated or perfluoro”* and restricting to environmental sciences, toxicology, or public, environmental, and occupational health categories. This search was further refined using the search terms “immun*” and “child*”

By about 2000, the widespread occurrence and persistence of PFASs in the environment became known [7], as reflected also by the presence of PFASs in serum samples from blood banks [16]. Only after this time, and especially during the most recent 10 years, did the scientific literature on PFASs expand (Fig. 1) [17]. Immune system deficits in PFOA-exposed mice were at first observed in studies of peroxisome proliferator activation [18]. Later, experimental studies of PFOS showed reductions in lymphoid cell numbers and de novo antibody synthesis [19], and a study in mice from 2009 demonstrated that PFOS exposure reduced the survival after influenza A infection [20]. Then followed in vitro evidence of adverse effects in human white blood cells [21]. Although the 1978 monkey study [5] could have been obtained from the U.S. EPA, none of these studies referred to these original findings.

Important evidence emerged after the discovery of PFAS contamination in the Mid-Ohio River Valley and the court-mandated health examinations [22]. In regard to immunotoxicity, an interim report showed that increased PFOA exposure was associated with changes in serum concentrations of immunoglobulins [23]. A more focused study determined antibody responses to flu vaccination [24]. Elevated serum-PFOA concentrations were associated with a reduced antibody titer rise, particularly to an A influenza virus strain, with an increased risk of not attaining the antibody level needed to provide long-term protection. A later study on 12 adult volunteers with background exposures showed that two of the subjects failed to respond to a tetanus-diphtheria booster and that the steepness of the antibody responses was negatively associated with the serum-PFAS concentrations [25]. Cross-sectional data have also suggested lower vaccination antibody concentrations at elevated background PFAS exposures [26].

The first prospective study assessing children’s antibody responses to routine childhood immunizations reported in 2012 that a doubling in exposure to PFOS and PFOA was associated with an overall decrease by up to 50% in the specific vaccine antibody concentration [27, 28]. When mutually adjusted, the regression coefficients for PFOA and PFOS changed only little [27]. Booster vaccine responses in children at age 5 years were lower at elevated serum-PFAS concentrations [28, 29]. A smaller Norwegian study of about 50 children aged 3 years also showed tendencies toward lower vaccination antibody concentrations at higher exposures during pregnancy [30]. As PFASs are now known to be transferred to the infant via human milk [31], it seems likely that PFAS exposures in early infancy represent a particular hazard to the adaptive immune system [32]. If true, the routine modeling of lifetime exposures for risk assessment is inappropriate, as it ignores the presence of vulnerable time windows.

PFAS exposure can also impact the body’s ability to fight off common infections, such as colds and gastroenteritis, as seen in the Norwegian study [30]. A larger, prospective study in Denmark found that increased maternal serum concentrations of PFOA and PFOS were significantly associated with a higher frequency of fever and symptoms in the children [33], in agreement with a subsequent study from Japan that relied on retrospective assessment of the disease incidence [34]. In contrast, a substudy from the Danish National Birth Cohort examined the hospitalization rates for a variety of infections, such as airway infection, middle ear infection, and appendicitis, through to age 11 years and showed no association with PFOS and PFOA in early pregnancy serum from the mother [35]. However, a recent report from the project team raised doubt about the validity of the PFAS analyses [36].

## Delayed interventions

Despite the support from both experimental and epidemiological data [37], most regulatory risk assessments of PFASs have focused on other target organs and have emphasized toxicity testing in rodents [4]. The first opinion from the European Food Safety Authority (EFSA) in 2009 [38] listed a single report on immunotoxicity under “Other endpoints”. That same year, the EPA issued provisional health advisories and concluded that “epidemiological studies of exposure to PFOA and adverse health outcomes in humans are inconclusive at present” [39]. Neither report referred to the 1978 monkey study that had become available in 2000. Early and more recent guidelines and recommended limits for PFOS and PFOA are shown in Table 2.

**Table 2:** Guideline values expressed in terms of acceptable concentrations of PFOS and PFOA in drinking water (ng/L),^a^ as compared with the estimated limit based on benchmark dose calculations for immunotoxicity in children [44]

Authority	Year	PFOS	PFOA
Australia			
	2016	70	560
Canada	2016	600	200
U.S. EPA	2009	200	400
	2016	70	70
ATSDR	2015	70	100
	2018	11	7
Minnesota	2008	300	300
	2017	27	35
New Jersey	2007	-	40
	2017	13	14
EFSA	2009	70	700
	2018	6.5	3
BMDL-based	2013	< 1	< 1

^a^Estimated from total intake limits, assuming 20% exposure contribution from water (rounded values)

The EPA prepared more detailed risk assessment reports for PFOA and PFOS in 2014 [40, 41]. These drafts conclude that the two major PFASs exhibit immunotoxicity in experimental models and that the epidemiological evidence is additive, although mixed exposures complicate the attribution of effects to specific PFASs. A similar conclusion was reached by an ATSDR ToxProfile on the perfluoroalkyls in 2015 [42]. The coverage of human immunotoxicity was very brief, and no mention of this potential was made in the sections on public health implications. Although the monkey studies were cited, the risk assessment reports did not refer to the 1992 study of exposure-associated immune cell abnormalities in workers.

More recently, the National Toxicology Program (NTP) in 2016 reviewed the immunotoxicity information on PFOS and PFOA and concluded that both are “presumed” to constitute immune hazards to humans [37]. The term “presumed” is the strongest below “known” in the NTP vernacular. Both PFASs suppress the antibody response in animal studies, while the evidence in humans is “moderate”, as all studies are observational (not experimental) and refer to mixed PFAS exposures. The revised ATSDR ToxProfile [43] just released concluded that decreased antibody response to vaccines is a potential outcome from exposure to all five PFASs commonly found in human blood samples. However, ATSDR stopped short of using epidemiology evidence for derivation of exposure limits.

Regulatory agencies frequently use benchmark dose calculations as a basis for generating exposure limits [38]. This approach relies on fitting a dose-response function to the data, and the benchmark dose (BMD) is defined as the dose that leads to a specific loss (or degree of abnormality) known as the benchmark response (BMR) in the outcome variable. The lower one-sided 95% confidence limit of the BMD is the benchmark dose level (BMDL), which is used as the point of departure for calculation of exposure limits. Relying on the vaccine antibody responses, BMDLs for PFOS and PFOA were calculated in 2013 to be about 1 μg/L serum [44], i.e., levels that are exceeded by a majority of the general population [45]. However, at first, these results were disregarded because of the absence of an unexposed control group [42], a condition that would be impossible to meet. Another concern was the high correlation between exposure components, such as PFOA and PFOS [40, 41, 43]. Still, mutual adjustment is possible and shows clear negative impacts of both of these major PFASs on immune system responses [27], and other calculations show virtually unchanged BMDLs for PFOA and PFOS after such adjustment [46].

In an updated opinion on PFOS and PFOA [47], EFSA used separate BMD calculations for several outcomes in humans, including immunotoxicity, relying on summary data in deciles or quartiles. For the vaccine response data [28], EFSA assumed that all subjects in the lowest decile exposure group had the same exposure, and the BMDs were similar to the average serum concentration in that group. For this reason, EFSA’s calculated BMDs are several fold higher than the ones obtained from the continuous dose-effect relationship [44]. Still, the new tolerable intake limits are substantially lower than other published guidelines (Table 2), though quite similar to the Minimal Risk Levels developed by ATSDR [43].

The “untested chemicals assumption”, as highlighted by the National Research Council [1] has clearly been inappropriately relied upon in past risk assessments of PFASs, and these substances must now be added to the list of environmental hazards [48] where standard risk assessment has failed. As a major reason, early evidence on PFAS toxicity was kept secret for 20 years or more, and even after its release, it was apparently overlooked. A related reason is the absence of academic PFAS research on the immune system and other sensitive target organs until about 10 years ago. Further, regulatory agencies relied on experimental toxicity studies and disregarded emerging epidemiological evidence. As a result, even some of the current guidelines are orders of magnitude above exposure levels at which associations with adverse effects have been reported.

The PFASs therefore constitute an unfortunate example that risk assessment may be inappropriate to assess human health risks from chemical exposures when crucial documentation has not yet been published. Recognizing the weaknesses of conventional risk assessment, scientists from the U.S. EPA recently recommended to consider the full range of available data and to include health endpoints that reflect the range of subtle effects and morbidities in humans [48]. The present summary of delayed discovery, dissemination and decision-making on the PFASs indicates that a more comprehensive assessment of adverse health risks is urgently needed and that PFAS substitutes, as well as other persistent industrial chemicals, should not be considered innocuous in the absence of relevant documentation [49].

## Conclusions

Early research on environmental PFAS exposures and their health implications became available at a substantial delay and was not taken into account in initial regulatory decisions on exposure abatement. Only in the last 10 years or so has environmental health research focused on the PFASs and revealed important human health risks, e.g., to the immune system. Although guideline values for PFASs in drinking water have decreased over time, they remain too high to protect against such toxicity. While the most commonly used PFASs will remain in the environment for many years, new PFAS substitutes are being introduced, although little information on adverse health risks is available. Given the serious delays in the discovery of PFAS toxicity, their persistence in the environment, and their public health impact, PFAS substitutes and other persistent industrial chemicals should be subjected to prior research scrutiny before widespread usage.

## Abbreviations

BMD: Benchmark dose
BMDL: Benchmark dose level
BMR: Benchmark response
EFSA: European Food Safety Authority
EPA: Environmental Protection Agency
NTP: National Toxicology Program
PFAS: Perfluorinated alkylate substance
PFOA: Perfluorooctanoic acid
PFOS: Perfluorooctanoic sulfonic acid
